# Neurobiological Pathways Linking Compulsive Sexual Behavior Disorder and Psychiatric Comorbidities: A Narrative Review

**DOI:** 10.7759/cureus.91966

**Published:** 2025-09-10

**Authors:** Agata Puszcz, Jan Górski, Weronika Pierudzka

**Affiliations:** 1 Family Medicine, University Clinical Hospital, Poznań, POL; 2 Hospital Medicine, University Clinical Hospital, Poznań, POL

**Keywords:** hypersexuality disorder, psychosexual disorders, sex addictions, sexual compulsiveness, sexual dysfunction

## Abstract

Compulsive sexual behavior disorder (CSBD) is a newly classified clinical condition characterized by persistent difficulty in controlling sexual impulses, repetitive sexual activity despite adverse consequences, and significant distress or impairment in various life areas. This narrative review explores the clinical and neurobiological overlaps between CSBD and selected mental disorders, including mood and anxiety disorders, attention-deficit/hyperactivity disorder, autism spectrum disorder, obsessive-compulsive disorder, and specific personality disorders.

The review emphasizes shared mechanisms, such as dysregulation of the dopaminergic reward system, impairments in emotional regulation, and altered connectivity in brain regions responsible for impulse control and affect processing. Structural and functional neural changes associated with impulsivity, such as abnormalities in the prefrontal cortex, orbitofrontal cortex, and limbic system, are discussed as potential contributors to both CSBD and its comorbid conditions. These findings support a dimensional approach to diagnosis and treatment, highlighting the need for integrated clinical strategies that account for overlapping symptoms and underlying vulnerabilities.

## Introduction and background

Compulsive sexual behavior disorder (CSBD) was first included in the 11th edition of the International Classification of Diseases (ICD-11), which has been in effect since 2022, despite earlier calls from researchers for the formulation of official diagnostic criteria more than a decade before. For many years, CSBD was referred to by various terms, such as sex addiction, hyperlibido, sexual impulsivity, problematic pornography use, or masturbation addiction. In 2010, Kafka introduced the term "hypersexuality," which was intended to be included in the Diagnostic and Statistical Manual of Mental Disorders (DSM)-5 classification, but this idea was abandoned due to a lack of sufficient scientific evidence to explain the mechanisms of this disorder [1,2].

The long-standing discussion regarding the conceptualization of CSBD resulted in various scientific positions concerning its nature and mechanisms. It was proposed to classify it as a sexual drive disorder, a behavioral addiction, an impulse control disorder, or a form of obsessive-compulsive disorder. Ultimately, CSBD was classified as an impulse control disorder, with diagnostic criteria including difficulties in controlling sexual impulses, excessive engagement in sexual activity, continuation of behaviors despite negative consequences, deterioration in social, professional, and personal functioning, and persistence of symptoms for at least six months. It should be differentiated from high libido, paraphilias, and hypersexuality occurring within the framework of other mental disorders, such as manic episodes or attention-deficit hyperactivity disorder (ADHD). CSBD represents an independent diagnostic entity characterized by specific criteria and the persistence of symptoms over time [1].

This review aimed to analyze the comorbidity of CSBD with other disorders and identify potential shared neurobiological and psychological mechanisms underlying this comorbidity. Such an analysis is clinically significant, allowing for a better assessment of whether pathological hypersexuality results from the overlap of CSBD with another disorder or constitutes an element of an underlying disease. Understanding comorbidity is crucial for more personalized patient treatment. The literature search was conducted using the PubMed, Scopus, Google Scholar, and Web of Science databases, including publications available up to March 2025. Inclusion criteria encompassed original research articles, review papers, and meta-analyses published in English or Polish. Case reports, mini-reviews, and publications older than 10 years were excluded from this analysis. The search terms included “CSBD” (for studies published from 2022 onward, following the introduction of ICD-11), as well as “CBD,” “hypersexuality,” and “problematic porn use” for studies published prior to 2022.

Due to the new approach to CSBD and the abandonment of equating this disorder with behavioral addiction, we decided to exclude comorbidity and shared mechanisms with addictions from this publication. Thus, we focus on compulsive sexual behavior primarily in the context of impulse control disorder, to which certain personality traits and a specific neurobiological phenotype predispose individuals.

## Review

Affective disorders

The relationship between CSBD and mood disorders is well-documented, with a comorbidity prevalence ranging from 36-81% [3]. Mood disorders coexisting with CSBD can manifest in various forms, including depression, dysthymia, and bipolar affective disorder (BPAD) [3,4].

Lew-Starowicz et al. suggest that deficient emotional self-regulation (DESR) may serve as a key link connecting mood disorders with compulsive sexual behaviors (CSB). Emotional dysregulation manifests as intense and difficult-to-control affective states, most commonly in the form of anger, sadness, or anxiety, characterized by increased frequency and prolonged duration compared to the general population. Individuals with DESR often employ maladaptive emotional regulation strategies, including self-destructive behaviors, substance abuse, and acts of aggression. Researchers highlight that compulsive sexual behaviors may also represent an acquired, uncontrolled coping mechanism for negative emotional states in this patient group [5].

In the proposed definition of hypersexuality for the Diagnostic and Statistical Manual of Mental Disorders (DSM-5), the following criteria related to emotional dysregulation were included: engaging in sexual activity in response to a dysphoric mood or stressful events, as well as unsuccessful attempts to control or reduce the frequency of sexual behaviors. Ultimately, this diagnosis was not incorporated into the classification [1].

Depression

Depression is a heterogeneous disorder that significantly impacts emotional and cognitive processes, leading to DESR, impaired memory, concentration, and negative information processing. Executive functions, including planning and decision-making, are also often disrupted in depression [6]. The neurobiological mechanisms of depression leading to DESR and reward system dysfunctions appear to be key in the co-occurrence of CSBD and depression and will be discussed below.

Studies on individuals with depression indicate several brain areas where neurotransmission disorders, particularly dopaminergic and serotonergic, may lead to DESR and thus explain the occurrence of CSBD and depression [4]. The most significant dysfunctions are considered to occur in the prefrontal cortex (PFC), particularly the orbitofrontal cortex (OFC), the amygdala, the anterior cingulate cortex (ACC), and the nucleus accumbens (NAc) [7].

Serotonergic and dopaminergic neurotransmission disorders observed in depression - particularly within the OFC - along with altered functional connectivity between the OFC and other brain regions such as the amygdala and striatum, may impair impulse control. This impairment can lead to a loss of behavioral regulation, including sexual behaviors, thereby contributing to the development of compulsive sexual behavior disorder (CSBD) [8].

Dopamine transmission disorders in the NAc, a central component of the reward system, may result in a reduced ability to experience pleasure and promote the search for immediate gratification through sexual behaviors, which appears to be a compensatory mechanism allowing for mood regulation and achieving satisfaction [9,10]. This suggests that CSBD may serve as a learned coping mechanism for negative emotions and stress in the course of depression [3,4].

Some sources also suggest a possible influence of hypothalamic-pituitary-adrenal (HPA) axis dysfunction on the development of CSBD in patients with depression. Chronic activation of the HPA axis due to prolonged stress leads to adaptive changes in serotonergic and dopaminergic systems, which may increase impulsivity and reduce the ability to control impulsive behaviors, including sexual ones [6].

Moreover, despite the frequently observed decrease in libido among depressed individuals, there are reports that a significant proportion of people with depression (15-25%) paradoxically experience increased sexual arousal [5]. The underlying mechanism of this sexual paradox is the aforementioned compensatory mechanism. Sexual activity allows for mood regulation and emotional tension reduction, providing personal contact with another person and positively influencing self-esteem, which may contribute to the occurrence of CSBD in some patients [4,5,11].

Given the information above, the primary mechanism responsible for the co-occurrence of depression and CSBD is considered to be the limited ability to control behavioral responses associated with the discussed neurobiological mechanisms underlying both disorders. Changes accompanying depression, such as weakened control and difficulties in emotional regulation, as well as increased impulsivity, create favorable conditions for the development of CSBD. Furthermore, sexual behaviors often serve as a tool for short-term emotional regulation in individuals with depression. Engaging in sexual activities can be perceived as a strategy to avoid negative emotions, additionally triggering a temporary feeling of relief associated with dopamine release in the reward system, reinforcing such behavior. Repeating sexual behaviors for self-regulation strengthens impulsive patterns while perpetuating an avoidance schema for negative emotions, instead of effectively processing them, deepens the interaction between these disorders [5].

Bipolar affective disorder

One of the defining symptoms of mania is the tendency to engage in pleasurable activities regardless of potentially dangerous consequences [12]. The combination of mania symptoms, including euphoria, grandiosity, excessive sexuality, and impulsivity, often leads individuals to engage in risky sexual behaviors [11]. Increased impulsivity in BPAD is present not only during manic episodes but also in euthymic and depressive states [12]. In BPAD, abnormalities are observed in the structure and functioning of brain areas responsible for emotion regulation and impulse control. The neurobiological mechanisms of impulsivity and impulsive sexual behaviors in BPAD are complex [12].

One of the most commonly described pathomechanisms of impulsivity, which may explain the relatively frequent co-occurrence of CSBD and BPAD, is the dysregulation of dopaminergic neurotransmission within the PFC, especially the OFC and the associated limbic system [13,14]. Studies indicate that abnormalities in this area may affect not only impulsivity but also risk assessment, decision-making, and reward system processing [7,13,14]. Many other sources describe the existence of dysfunctions in OFC functioning and the reward system in CSBD [4,7,10]. In the context of reward system dysfunctions, Büchel et al. highlight the role of reduced activation of the NAc in reward anticipation in BPAD, which may lead to compulsive reward-seeking, manifesting as the occurrence of CSBD [9].

Moreover, many studies indicate that CSBD is associated with altered functioning of brain areas involved in habits, impulse control, and reward processing, such as the frontotemporal cortex (FTC), amygdala, and ventral striatum (VStr). Scientists also emphasize that the brain's reward system plays a key role in CSBD, and its underlying mechanisms are similar to those found in behavioral addictions [10,14]. The cited studies suggest that impulsivity and compulsivity in BPAD may be more strongly associated with CSBD than with other forms of addiction, indicating the specificity of this relationship.

Anxiety disorders

People with anxiety disorders exhibit a significantly higher risk of comorbid mental illnesses, including sexual addiction [15]. The reported co-occurrence rates of CSBD with anxiety disorders range from 46-96%, suggesting a significant link between these conditions [3]. The shared neurobiological and psychological basis of both disorders is related to difficulties in emotion regulation and impulse control and constitutes a key pathomechanism for both conditions. Among anxiety disorders most commonly associated with CSBD are generalized anxiety disorder (GAD) and social anxiety disorder (SAD) [16].

At the neurobiological level, the frequent co-occurrence of anxiety disorders and CSBD can be explained by dysfunctions in the limbic system, particularly within the amygdala, hippocampus, and cingulate gyrus, as well as related structures, such as the PFC and VStr [14,17]. These areas are part of the reward system, linked to the mesolimbic dopaminergic pathway, and are responsible for processing emotions, impulses, and reward-seeking behavior. Studies indicate that in anxiety disorders, such as GAD and SAD, there is increased neuronal activity in the amygdala and insula, structures involved in recognizing and processing emotionally negative stimuli [18]. Hyperactivity in these brain regions may lead to heightened emotional reactivity and a predisposition to CSBD as a mechanism for coping with anxiety.

It is believed that a similar effect may be generated by dysfunctions in the cingulate gyrus, particularly in its dorsal anterior part (dACC), where hyperactivity may impair the regulation of impulsive behaviors and result in excessive reward-seeking, thereby contributing to the development of CSBD [10,14].

Reduced PFC activity, characteristic of anxiety disorders, may also limit the ability to control emotional responses to negative stimuli and lead to the development of increased impulsivity, typical of CSBD [13]. Studies suggest that patients with GAD also exhibit cognitive and executive function deficits, which exacerbate DESR and further impair impulse control [14].

Additionally, dysregulation of the HPA axis, involving disturbances in cortisol release in response to chronic stress and anxiety, may lead to structural and functional changes in the limbic system, thereby exacerbating DESR observed in CSBD [5]. Changes in dopaminergic and serotonergic system function also contribute to these disorders, increasing the need for gratification while weakening impulse control, which fosters the co-occurrence of the aforementioned conditions [17].

Attention-deficit hyperactivity disorder

ADHD is a heterogeneous neurodevelopmental disorder initiated in early childhood, which often accompanies patients into adult life. The core symptoms of the disease include attention deficit, motor hyperactivity, and impulsivity. Due to the novelty of the concept of CSBD in academic literature, there is a lack of data regarding the comorbidity of ADHD with CSBD. Nevertheless, in the summary of his scientific works, Kafka described that the comorbidity of ADHD with hypersexuality ranges between 17% and 19%. This is an older concept that existed before CSBD but meets many of the same diagnostic criteria [2].

Studies suggest that the symptomatology of ADHD plays a determining role in the comorbidity of both disorders. Hyperactivity, impulsivity, and attention deficit in ADHD are associated with the occurrence of risky sexual behaviors and symptoms of hypersexuality. This relationship was confirmed by the studies by Bőthe et al. and Doroldi et al. [19,20].

Analyzing the mechanisms of co-occurrence of ADHD and CSBD, particular attention was drawn to dysfunctions of the dopaminergic reward system. Dysfunction in the form of dopamine deficiency predisposes to the development of reward deficiency syndrome (RDS), a syndrome characterized by the inability to derive pleasure or experience reward from daily activities. This disorder is the cause of so-called dopamine-seeking behavior, which includes CSB [21].

Moreover, dysfunction of the dopaminergic pathway within the PFC results in stronger reinforcement of stimuli in a patient with ADHD compared to a neurotypical person. The combination of these two factors creates conditions that allow for the formation of pathological couplings within the reward system, predisposing a person with ADHD to the development of psychological and physical addictions [22]. The above-mentioned claims regarding dopaminergic hypoactivity in ADHD align with current knowledge, which describes dopaminergic hypoactivity in the frontostriatal system [23].

Perrota described the role of the ventral tegmental area, NAc, amygdala, basal ganglia, PFC, and OFC in the mechanisms contributing to behavioral and physical addictions, as well as the development of CSB [24]. Dysfunction within shared structures may explain the comorbidity of both conditions.

Moreover, Draps et al. demonstrated a significant decrease in fractional anisotropy in cerebellar tracts in CSBD [25]. Fractional anisotropy is a quantitative indicator of white matter integrity in neurological studies, and its decrease indicates microstructural abnormalities in white matter. Similar observations were noted in the case of ADHD, where Parkkinen et al. documented a decrease in fractional anisotropy in the middle cerebellar peduncle. However, effective pharmacotherapy with methylphenidate increased fractional anisotropy in the inferior cerebellar peduncle [26]. The presented data confirm the co-occurrence of white matter microstructural abnormalities in the cerebellum in both conditions. This is significant because it may contribute to the development of impulsivity and hyperactivity and, thus, indirectly to the development of CSBD [27].

An analysis of the pathomechanisms and structural disorders of the CNS occurring in ADHD indicates the crucial role of dopaminergic system dysfunction in the development of ADHD and CSBD comorbidity. Structural and functional disorders concerning components of the reward system result in dopaminergic hypoactivity and contribute to the development of RDS and impulsivity, indirectly leading to the development of CSBD. The next potential step in exploring the mechanisms of ADHD and CSBD comorbidity would be to conduct identical studies for both conditions, as their absence hinders direct comparison. Additionally, an interesting area for investigation is the impact of methylphenidate therapy on the fractional anisotropy of cerebellar tracts in individuals with CSBD to compare treatment outcomes with those obtained in individuals with ADHD.

Autism spectrum disorders

ASD is a developmental disorder that affects verbal and non-verbal communication, social interactions, behavior, and play. Schöttle et al. found that the comorbidity of ASD with CSBD is 30.4% in men and 10.0% in women [28]. A typical feature of ASD is the routine repetition of behaviors, which, during adolescence and adulthood, can manifest as erotic behaviors. Moreover, sensory disturbances commonly found in ASD can lead to either hyporeactivity or hyperreactivity to sexual experiences [29]. Consequently, individuals with ASD may be predisposed to developing hypersexuality or problematic sexual behaviors. A study conducted by Fernandes et al. reported that 24% of individuals with high-functioning ASD engage in paraphilic sexual fantasies, including pedophilia, voyeurism, or sadomasochism [30]. When analyzing the pathogenesis of ASD in the context of comorbidity with CSBD, special attention has been given to dysfunctions within the dopaminergic reward system.

Similarities in the dysfunctions of the dopaminergic system in ASD and CSBD have been mainly observed within the mesocorticolimbic pathway, which is responsible for regulating emotions, impulsivity, motivation, and reward processing. In ASD, dysregulation in dopamine levels within the mesocorticolimbic pathway and reduced dopaminergic activity in the PFC and NAc have been observed [31]. These dysfunctions can lead to difficulties in emotional regulation, decreased sensitivity to social stimuli, motivational problems, impulsivity, and reduced responsiveness to rewarding stimuli, which may explain the increased need for intensely stimulating sexual experiences.

Similar phenomena have been observed in studies on CSBD, where Kowalewska et al. demonstrated a link between abnormalities in structures within the mesocorticolimbic pathway and the occurrence of CSBD [10]. In the context of sexual behaviors, structural similarities between ASD and CSBD may result in similar behavioral disturbances and explain their comorbidity.

In conclusion, the primary mechanism contributing to the comorbidity of ASD and CSBD appears to be the dysfunction within the dopaminergic reward system, leading to behavioral disturbances and altered responses to sexual stimuli. Despite numerous studies confirming differences in the sexuality of individuals with ASD, the underlying mechanisms remain not fully understood.

Obsessive-compulsive disorder

Studies indicate that 5-7% of individuals with OCD may develop CSBD during their lifetime, with 75% of cases involving men [32]. A potential cause of CSBD co-occurrence in OCD patients is the presence of obsessive thoughts of a sexual nature, which affect up to 25% of individuals with OCD [33]. Individuals with OCD tend to exhibit compulsive sexual behaviors as a way of coping with anxiety and negative emotional states [32,33].

Dysfunctions within the PFC and basal ganglia structures, particularly in the caudate nucleus responsible for reward processing, may play a key role in the pathogenesis of OCD and CSBD comorbidity [10,34]. Neuroimaging studies in both OCD and CSBD patients reveal increased activity in corticostriatal structures such as the OFC, ACC, and caudate nucleus, which translates to difficulties in impulse inhibition and a tendency toward compulsive behaviors [7,14,34]. In both disorders, studies indicate increased OFC activity, particularly in the context of reward anticipation and in response to sexual stimuli, hyperactivity of the striatum, including the caudate nucleus, and ACC [10]. This may lead to excessive engagement in sexual behaviors as a form of reward [13].

In addition to structural changes, serotonin deficiency in the OFC and basal ganglia occurs in both disorders, which also promotes increased impulsivity and a higher likelihood of developing compulsive behaviors [7,34]. In OCD, excessive dopaminergic activity in the striatum intensifies avoidance responses in affected individuals, resulting in an increased tendency to perform compulsions as a defense mechanism against anticipated negative consequences. Similarly, hyperactivity of the mesocorticolimbic dopaminergic pathway reinforces compulsive behaviors aimed at reducing anxiety, giving them characteristics of behavioral addiction [34]. In the case of CSBD, mesocorticolimbic dopaminergic hyperactivity may amplify the craving for sexual stimuli [14]. Additionally, the literature highlights the clinical complexity of coexisting OCD and CSBD, suggesting that both conditions may mutually exacerbate each other, necessitating more complex therapeutic interventions for affected patients [32].

Personality disorders

Pathological personality is a collection of dysfunctional traits and rigid behavior patterns. Many research teams have attempted to link these rigid behavioral patterns (and, in a broader sense, specific personality disorders) to the occurrence of CSBD. One of the first teams to study the co-occurrence of personality disorders with CSBD was Raymond et al. The study utilized the Structured Clinical Interviews for Personality Diagnosis (SCID-P) and SCID-II questionnaires, as well as the compulsive sexual behavior inventory (CSBI) to diagnose compulsive sexual behaviors. Among the studied group, co-occurrence with CSB was found in 46% of patients with any personality disorder and 38% of those with any impulse control disorder. However, it should be noted that the study was conducted before the official diagnostic criteria for CSBD were formulated, meaning that participants who did not meet the current diagnostic guidelines for CSBD may have been included in the experiment [35]. Additionally, the SCID-P questionnaire relies on interviews, making it a subjective tool that heavily depends on the experience and judgment of the diagnostician. The lack of objective measures means that many individuals surveyed in the abovementioned study may not have had a personality disorder. Despite its weaknesses, Raymond's study paved the way for future research.

According to a survey conducted by Jepsen and Brzank, the personality traits most strongly correlated with CSBD were impulsivity (associated with ASPD), narcissism, and histrionic tendencies. The researchers used the Narcissistic Personality Inventory (NPI), Barrett Impulsiveness Scale (BIS), and Self-Esteem Scale [36].

The co-occurrence of CSBD and narcissistic traits was also confirmed by Volkert et al. However, it is necessary to distinguish between the comorbidity of these two disorders and the rigid sexual behavior patterns embedded in narcissistic personality disorder (sexual narcissism). Narcissistic patients often have irrational beliefs about sex, spend a lot of time watching pornography and masturbating, experience reduced pleasure from partnered sex, and consequently devalue their partner. Moreover, even in cases of comorbid NPD and CSBD, this group of patients may not experience the distress or suffering necessary for a CSBD diagnosis due to the general difficulty narcissists have in gaining insight into their emotions [37,38]. Among narcissism researchers, there is no consensus on whether compulsive sexual behaviors are always a component of the disorder or if they indicate an additional CSBD diagnosis.

Brain regions associated with social cognition, such as the medial prefrontal cortex (MPFC), may play a role in shaping narcissism-related sexual behaviors, particularly in the context of interpersonal interactions. Mao et al. demonstrated that the Pathological Narcissism Inventory (PNI) score negatively correlates with cortical thickness in the MPFC, as measured using surface-based morphometry. Klucken et al. found that reduced MPFC connectivity with the caudate nucleus may underlie CSBD. However, research on the neurobiology of sexual narcissism is still in its early stages, and further studies are needed to better understand the links between brain function and this personality disorder [39-41].

The findings on the co-occurrence of CSBD and ASPD are inconclusive, even though impulsivity is a well-researched predisposing factor [1,42,43]. Impulsivity and sensation-seeking are often linked to criminal and risky behaviors. Much of the knowledge about ASPD and its comorbidity comes from studies conducted in correctional settings. According to available data, 20-70% of the prison population meets the diagnostic criteria for antisocial personality disorder (ASPD), making them a suitable research cohort for studying this condition [44]. Research has shown a statistically significant co-occurrence of ASPD with paraphilic disorders and sexual sadism [45]. It was suspected that the high libido observed in this group, along with a high level of impulsivity, could imply compulsive sexual behaviors.

Sindermann et al. examined the possible comorbidity of CSBD with the "dark triad of personality" and used the Short Dark Triad Questionnaire for this purpose. The study found statistical significance only among women with psychopathic traits. Additionally, in the case of ASPD patients who were convicted of sexual offenses and whose crimes were characterized by lower organization and greater impulsivity, the CSBD criteria were more frequently met [46].

Other studies on prisoners convicted of sexual offenses were conducted by Efrati et al. The researchers used Young’s Early Maladaptive Schema Questionnaire (YSQ). The hypothesis was that maladaptive schemas in sexual offenders would correlate with CSBD. However, the study found that CSBD was more common among non-offending sex addicts anonymous (SAA) members than among sexual offenders [47]. Another team, Ryan et al., examined 293 men convicted of sexual offenses in terms of general impulsivity and sensation-seeking, which were not found to be statistically significant. It can, therefore, be concluded that the type of impulsivity associated with aggression and more typical of ASPD is different from the impulsivity studied in the context of CSBD. Impulsivity and dopamine-seeking behaviors are well-studied correlates of CSBD in the general population, but not in correctional populations [47,48].

Apart from ASPD, another personality disorder strongly associated with impulsivity is BPD. An analysis conducted by Ballester-Arnal et al. found that in a group of CSBD patients, the prevalence of BPD was 5.9% [49]. The research team in the study by Jardin et al. suggested that CSB may serve as a coping mechanism for intense emotions in individuals with BPD [50].

Furthermore, it is suggested that the statistically significant co-occurrence of BPD and CSBD may result from dysregulation within the PFC and limbic system, which are crucial in the pathogenesis of these disorders when they occur separately. It is speculated that hyperactivity of the amygdala and insula during emotional stimulus processing, along with the reduced activity of the OFC and dACC, is responsible for CSB as a consequence of insufficient emotional regulation by control centers. Dysregulation of the reward system may also explain this comorbidity, as difficulties in delaying gratification are a predisposing factor for CSBD and problematic pornography use [11,13].

Research on the comorbidity of HPD and CSBD is still limited. Most of the data comes from a study by Hughes et al., which focused exclusively on women. The study used the CSBI scale, Steven E. Hyler's Personality Disorder Questionnaire (PDQ-4) in its histrionic and narcissistic subscales, and the Postrefusal Sexual Persistence Scale (PSP) to measure the use of sexual coercion. Both the use of emotional manipulation and deception to pressure a partner, as well as exploiting their intoxicated state, were positively correlated with both sexual compulsivity and histrionic traits [51]. All relevant information found regarding the neuropathogenesis of CSBD is listed in Table 1.

**Table 1:** Neuropathogenesis of CSBD. OFC: orbitofrontal cortex; PFC: prefrontal cortex; HPA: hypothalamic-pituitary-adrenal; PFC: prefrontal cortex; VStr: ventral striatum; CSBD: compulsive sexual behavior disorder; dACC: dorsal anterior part

Pathomechanism	Clinical ramifications	Source
Disturbances in dopamine neurotransmission in the NAc	Behavioral control disorders, reinforcement of compulsive behavior patterns, reduced ability to experience pleasure, and compulsive reward-seeking	Büchel et al. [9]
Disruptions in 5-HT and dopamine neurotransmission in the PFC, especially in the OFC	Limited ability to control emotional responses and impulsivity	Mavrogiorgou et al. [7]
Changes in the functional connectivity of the OFC with the limbic system and the striatum	Impulse and habit control disorders, emotional dysregulation, impaired risk assessment, and information processing disorders within the reward system	Mavrogiorgou et al. [7]
Dysfunction of the ventral tegmental area, NAc, amygdala, basal ganglia, PFC, and OFC	Predisposition to behavioral and physical addictions	Perrota [24]
Decrease in fractional anisotropy in cerebellar pathways	Hyperactivity and impulsivity	Draps et al. [25]
Disruptions in the mesocorticolimbic pathway	Limited ability to regulate emotions, reduced sensitivity to social stimuli, motivational problems, impulsivity, and decreased response to rewarding stimuli	Kowalewska et al. [10]
Disruptions in the HPA axis	Dopaminergic and serotonergic dysregulation, impulsivity, structural and functional changes in the limbic system	Lew-Starowicz et al. [5]
Hyperactivity of the PFC-VStr-amygdala system	Increased emotional reactivity, impulsive behaviors, and reward-seeking	Voon et al. [14]
Hyperactivity of the dACC	Impaired regulation of impulsive behaviors and excessive reward-seeking	Kowalewska et al. [10]

## Conclusions

The primary aim of this analysis was to review the neurobiological and psychological mechanisms underlying compulsive sexual behavior disorder (CSBD) and their role in explaining its comorbidity with other disorders. Dysfunctions in dopaminergic transmission within the nucleus accumbens (NAc), implicated in addictions and affective disorders, suggest CSBD’s addictive nature and its classification as a behavioral addiction. This dysfunction may also account for dysphoria and helplessness in patients. From the perspective of emotional self-regulation, CSBD may emerge as a consequence of affective or anxiety disorders; conversely, its progression could exacerbate neural dysregulation, increasing vulnerability to depression. In affective disorders, CSBD might function as a primary disorder, a secondary phenomenon, or a compensatory response. Similarly, serotonergic and dopaminergic dysregulation in the prefrontal cortex, particularly in the orbitofrontal cortex (OFC), is central to obsessive-compulsive disorder (OCD), where CSBD symptoms may present as obsessions or compulsions. This overlap supports theories positioning CSBD as a sexually-oriented subtype of OCD or as a condition developing secondary to OCD.

Impulsivity has been identified as a predisposing trait for CSBD, with neuroimaging studies indicating reduced fractional anisotropy in cerebellar tracts, suggesting compromised white matter integrity and impaired emotional regulation. These findings support conceptualizing CSBD as an impulse control disorder, though such anomalies may also relate to preexisting conditions like ADHD. The OFC’s involvement in erotic stimulus regulation and the striatum’s role in reward processing imply that disrupted connectivity between these structures in CSBD contributes to maladaptive sexual motivation. These disruptions may also extend to affective and anxiety symptoms, reflecting broader behavioral dysregulation. Ultimately, alterations in OFC-striatum-limbic connectivity may stem from CSBD or represent a predispositional factor for multiple disorders, underscoring the potential for shared neurobiological predictors rather than direct causality. Despite being a newly classified diagnosis, CSBD's clinical relevance is increasingly acknowledged. Notably, many affected individuals avoid sexology specialists, highlighting the need to raise awareness among general medical and mental health practitioners. Future research should aim to clarify the causal direction of the observed neurobiological alterations, distinguishing between changes that represent predispositional vulnerabilities and those that arise as consequences of CSBD. Longitudinal neuroimaging studies may provide greater insight into the developmental pathways of CSBD and its relationship with comorbid conditions such as affective, anxiety, and impulse control disorders. Moreover, investigating sex- and gender-related differences, genetic underpinnings, and the role of environmental stressors could contribute to a more nuanced understanding of etiological pathways. Lastly, translational studies linking neurobiological findings with treatment outcomes are needed to inform evidence-based interventions and guide the development of novel therapeutic approaches.

## References

[1] Gola M (2022). Rozwój wiedzy na temat kompulsywnych zachowań seksualnych - autoreferat Mateusz Gola. [Preprint]. PsyArXiv.

[2] Kafka MP (2010). Hypersexual disorder: a proposed diagnosis for DSM-V. Arch Sex Behav.

[3] Briken P, Bőthe B, Carvalho J (2024). Assessment and treatment of compulsive sexual behavior disorder: a sexual medicine perspective. Sex Med Rev.

[4] Görts P, Savard J, Görts-Öberg K (2023). Structural brain differences related to compulsive sexual behavior disorder. J Behav Addict.

[5] Lew-Starowicz M, Lewczuk K, Nowakowska I, Kraus S, Gola M (2020). Compulsive sexual behavior and dysregulation of emotion. Sex Med Rev.

[6] Jiang Y (2024). A theory of the neural mechanisms underlying negative cognitive bias in major depression. Front Psychiatry.

[7] Mavrogiorgou P, Enzi B, Klimm AK, Köhler E, Roser P, Norra C, Juckel G (2017). Serotonergic modulation of orbitofrontal activity and its relevance for decision making and impulsivity. Hum Brain Mapp.

[8] Zheng KZ, Wang HN, Liu J (2018). Incapacity to control emotion in major depression may arise from disrupted white matter integrity and OFC-amygdala inhibition. CNS Neurosci Ther.

[9] Büchel C, Peters J, Banaschewski T (2017). Blunted ventral striatal responses to anticipated rewards foreshadow problematic drug use in novelty-seeking adolescents. Nat Commun.

[10] Kowalewska E, Grubbs JB, Potenza MN, Gola M, Draps M, Kraus SW (2018). Neurocognitive mechanisms in compulsive sexual behavior disorder. Curr Sex Health Rep.

[11] Trovão JN, Serefoglu EC (2018). Neurobiology of male sexual dysfunctions in psychiatric disorders: the cases of depression, anxiety, mania and schizophrenia. Int J Impot Res.

[12] Santana RP, Kerr-Gaffney J, Ancane A, Young AH (2022). Impulsivity in bipolar disorder: state or trait?. Brain Sci.

[13] Golec K, Draps M, Stark R, Pluta A, Gola M (2021). Aberrant orbitofrontal cortex reactivity to erotic cues in compulsive sexual behavior disorder. J Behav Addict.

[14] Voon V, Mole TB, Banca P (2014). Neural correlates of sexual cue reactivity in individuals with and without compulsive sexual behaviours. PLoS One.

[15] Gola M, Miyakoshi M, Sescousse G (2015). Sex, impulsivity, and anxiety: interplay between ventral striatum and amygdala reactivity in sexual behaviors. J Neurosci.

[16] Sassover E, Weinstein A (2020). Should compulsive sexual behavior (CSB) be considered as a behavioral addiction? A debate paper presenting the opposing view. J Behav Addict.

[17] Javanbakht A (2018). A theory of everything: overlapping neurobiological mechanisms of psychotherapies of fear and anxiety related disorders. Front Behav Neurosci.

[18] Saraff P, Shikatani B, Rogic AM, Dodig EF, Talluri S, Murray-Latin H (2023). Intolerance of uncertainty and social anxiety: an experimental investigation. Behav Change.

[19] Bőthe B, Koós M, Tóth-Király I, Orosz G, Demetrovics Z (2019). Investigating the associations of adult ADHD symptoms, hypersexuality, and problematic pornography use among men and women on a largescale, non-clinical sample. J Sex Med.

[20] Doroldi D, Jannini TB, Tafà M, Casale AD, Ciocca G (2024). ADHD and hypersexual behaviors: the role of impulsivity, depressive feelings, hypomaniacal symptoms and psychotic prodromes. J Affect Disord Rep.

[21] Febo M, Blum K, Badgaiyan RD (2017). Dopamine homeostasis: brain functional connectivity in reward deficiency syndrome. Front Biosci (Landmark Ed).

[22] Muldner-Nieckowski Ł, Jakima S, Bilejczyk A (2022). Hypersexuality. PZWL, Warsaw.

[23] Seifert R (2018). Basic Knowledge of Pharmacology.

[24] Perrota G (2020). Dysfunctional sexual behaviors: definition, clinical contexts, neurobiological profiles and treatments. IJSRHC.

[25] Draps M, Kowalczyk-Grębska N, Marchewka A, Shi F, Gola M (2021). White matter microstructural and compulsive sexual behaviors disorder - diffusion tensor imaging study. J Behav Addict.

[26] Parkkinen S, Radua J, Andrews DS, Murphy D, Dell'Acqua F, Parlatini V (2024). Cerebellar network alterations in adult attention-deficit/hyperactivity disorder. J Psychiatry Neurosci.

[27] Rashidi F, Parsaei M, Kiani I (2024). White matter correlates of impulsive behavior in healthy individuals: a diffusion magnetic resonance imaging study. PCN Rep.

[28] Schöttle D, Briken P, Tüscher O, Turner D (2017). Sexuality in autism: hypersexual and paraphilic behavior in women and men with high-functioning autism spectrum disorder. Dialogues Clin Neurosci.

[29] Parchomiuk M (2019). Sexuality of persons with autistic spectrum disorders (ASD). Sex Disabil.

[30] Fernandes LC, Gillberg CI, Cederlund M, Hagberg B, Gillberg C, Billstedt E (2016). Aspects of sexuality in adolescents and adults diagnosed with autism spectrum disorders in childhood. J Autism Dev Disord.

[31] Mandic-Maravic V, Grujicic R, Milutinovic L, Munjiza-Jovanovic A, Pejovic-Milovancevic M (2021). Dopamine in autism spectrum disorders - focus on D2/D3 partial agonists and their possible use in treatment. Front Psychiatry.

[32] Fuss J, Briken P, Stein DJ, Lochner C (2019). Compulsive sexual behavior disorder in obsessive-compulsive disorder: prevalence and associated comorbidity. J Behav Addict.

[33] Snaychuk LA, Ferrão YA, Fontenelle LF, Miguel EC, de Mathis MA, Scanavino MD, Kim HS (2022). Co-occurring obsessive-compulsive disorder and compulsive sexual behavior: clinical features and psychiatric comorbidities. Arch Sex Behav.

[34] Calzà J, Gürsel DA, Schmitz-Koep B, Bremer B, Reinholz L, Berberich G, Koch K (2019). Altered cortico-striatal functional connectivity during resting state in obsessive-compulsive disorder. Front Psychiatry.

[35] Raymond NC, Coleman E, Miner MH (2003). Psychiatric comorbidity and compulsive/impulsive traits in compulsive sexual behavior. Compr Psychiatry.

[36] Jepsen D, Brzank PJ (2022). Hypersexual behaviour among young adults in Germany: characteristics and personality correlates. BMC Psychiatry.

[37] McNulty JK, Widman L (2013). The implications of sexual narcissism for sexual and marital satisfaction. Arch Sex Behav.

[38] Volkert J, Gablonski TC, Rabung S (2018). Prevalence of personality disorders in the general adult population in Western countries: systematic review and meta-analysis. Br J Psychiatry.

[39] Draps M, Adamus S, Wierzba M, Gola M (2022). Functional connectivity in compulsive sexual behavior disorder - systematic review of literature and study on heterosexual males. J Sex Med.

[40] Klucken T, Wehrum-Osinsky S, Schweckendiek J, Kruse O, Stark R (2016). Altered appetitive conditioning and neural connectivity in subjects with compulsive sexual behavior. J Sex Med.

[41] Mao Y, Sang N, Wang Y (2016). Reduced frontal cortex thickness and cortical volume associated with pathological narcissism. Neuroscience.

[42] Perley-Robertson B, Helmus LM, Derkzen D, Serin RC (2016). Do sex offenders against adults, sex offenders against children, and non-sex offenders differ in impulsivity?. SOTP.

[43] Saladino V, Eleuteri S, Nuzzi A, Verrastro V (2023). Family attachment, sexuality, and sexual recidivism in a sample of Italian sexual offenders: preliminary results. Healthcare (Basel).

[44] Czubak K, Gawda B (2020). Occurrence of personality disorders among inmates and their social rehabilitation. Pol J Soc Rehabil.

[45] DeLisi M, Drury AJ, Caropreso D, Heinrichs T, Tahja KN, Elbert MJ (2018). Antisocial personality disorder with or without antecedent conduct disorder: the differences are psychiatric and paraphilic. Crim Justice Behav.

[46] Sindermann C, Sariyska R, Lachmann B, Brand M, Montag C (2018). Associations between the dark triad of personality and unspecified/specific forms of Internet-use disorder. J Behav Addict.

[47] Efrati Y, Shukron O, Epstein R (2019). Compulsive sexual behavior and sexual offending: differences in cognitive schemas, sensation seeking, and impulsivity. J Behav Addict.

[48] Ryan TJ, Huss MT, Scalora MJ (2017). Differentiating sexual offender type on measures of impulsivity and compulsivity. Sex Addict Compuls.

[49] Ballester-Arnal R, Castro-Calvo J, Giménez-García C, Gil-Juliá B, Gil-Llario MD (2020). Psychiatric comorbidity in compulsive sexual behavior disorder (CSBD). Addict Behav.

[50] Jardin C, Sharp C, Garey L, Vanwoerden S, Crist N, Elhai JD, Zvolensky MJ (2017). Compelled to risk: does sexual compulsivity explain the connection between borderline personality disorder features and number of sexual partners?. J Pers Disord.

[51] Hughes A, Brewer G, Khan R (2020). Sexual coercion by women: the influence of pornography and narcissistic and histrionic personality disorder traits. Arch Sex Behav.

